# Is it appropriate for Korean women to adopt the 2009 Institute of Medicine recommendations for gestational weight gain?

**DOI:** 10.1371/journal.pone.0181164

**Published:** 2017-07-13

**Authors:** Jeong ha Wie, In Yang Park, Jeong Namkung, Hae Won Seo, Min Jin Jeong, Ji Young Kwon

**Affiliations:** Department of Obstetrics and Gynecology, College of Medicine, The Catholic University of Korea, Seoul, Republic of Korea; Univesity of Iowa, UNITED STATES

## Abstract

**Background:**

The 2009 Institute of Medicine (IOM) guidelines for gestational weight gain (GWG) are intended for use among women in the United States. Little data are available on whether the 2009 IOM recommendations can be applied to Asian women. This study aimed to evaluate whether the recommendations are related to adverse pregnancy outcomes in Korean pregnant women.

**Methods and findings:**

A retrospective cohort study was conducted for all singleton-pregnant women at a university hospital in Korea. After classifying the enrolled women into four Korean pre-pregnancy body mass index (BMI) categories, the risk of adverse pregnancy outcomes were analyzed for women who gained inadequate or excessive GWG based on 2009 IOM recommendations. Of 7,843 pregnancies, 64.0% of women had normal pre-pregnancy BMI and 42.7% achieved optimal GWG. Across all BMI categories, adverse pregnancies outcomes such as small for gestational age (SGA), large for gestational age (LGA), preterm birth, preeclampsia, and cesarean due to dystocia were significantly associated with GWG (all *P* ≤ 0.001).Women with normal BMI who gained inadequate weight were more likely to develop SGA and preterm birth and less likely to develop LGA (adjusted odds ratio (aOR) 2.21, 1.33, and 0.54, respectively). Whereas, women with normal BMI who gained excessive weight were more likely to develop LGA, preterm birth, preeclampsia, and cesarean section due to dystocia (aOR 2.10, 1.33, 1.37, and 1.37, respectively) and less likely to develop SGA (aOR 0.60).

**Conclusions:**

It is tolerable for Korean women to follow recommended GWG from the 2009 IOM guidelines to decrease adverse pregnancy outcomes. This will be helpful for antenatal care on GWG not only for Korean pregnant women, but also other Asian women who have lower BMI criteria than Caucasian women.

## Introduction

Appropriate weight gain during pregnancy is very important to optimize pregnant women’s health and the health of her offspring. Many studies have shown that gestational weight gain (GWG) is related to preterm birth, low birth weight, macrosomia, and delivery of a small-for-gestational-age (SGA) or large-for-gestational-age (LGA) neonate. It is also well-established that cesarean delivery and postpartum weight retention are related to excessive GWG [1–4].

Many countries refer to the latest version of the Institute of Medicine (IOM) guidelines, published in 2009, for optimal GWG [5]. These guidelines are based on pre-pregnancy body mass index (BMI). According to the IOM guidelines, which use the World Health Organization (WHO) BMI categories, the optimal GWG is 12.5–18 kg, 11.5–16 kg, 7–11.5 kg, and 5–9 kg for underweight, normal weight, overweight, and obese women, respectively, during pre-pregnancy [5]. However, these recommendations are for various racial and ethnic women that comprise the US, and the applicability of these recommendations to populations outside the US is not clear [5]. Thus, evaluating whether these recommendations are significantly related to pregnancy outcomes to determine whether they are applicable to women of other races is necessary.

Asian women are particularly substantially shorter or thinner than women in the US. Asian women also have higher prevalence of type 2 diabetes and cardiovascular disease than Caucasian women of the same age and BMI [6, 7]. Therefore, in Korea, the obesity society states BMI classification was set to 23–24.9 kg/m^2^ for overweight and ≥ 25 kg/m^2^ for obesity, which is much lower than the WHO BMI cut-offs. As the IOM recommendations were determined based on pre-pregnancy BMI groups, it is also necessary to examine if the IOM recommendations can be applied to Asian women. A few studies on optimal GWG have been conducted in several Asian countries [8–12]. However, these previous studies had some limitations such as using WHO BMI categories for Caucasian populations [10, 12], presenting the new GWG guidelines with quartile values (25 – 75th percentile) [8, 9] or including small study groups [11]. Thus, additional studies on the optimal GWG for pregnant Asian women are needed.

This study examined adverse pregnancy outcomes associated with the 2009 IOM guidelines for GWG recommendations to verify whether they are valid for Korean women. Results from this study will be helpful in determining whether a new optimal GWG standard for Asians is needed.

## Materials and methods

This retrospective cohort study was conducted for all singleton-pregnant women who had given birth at ≥ 28 weeks of gestation at a tertiary university hospital in Seoul, Korea from 2000 to 2007.

**Fig 1:**
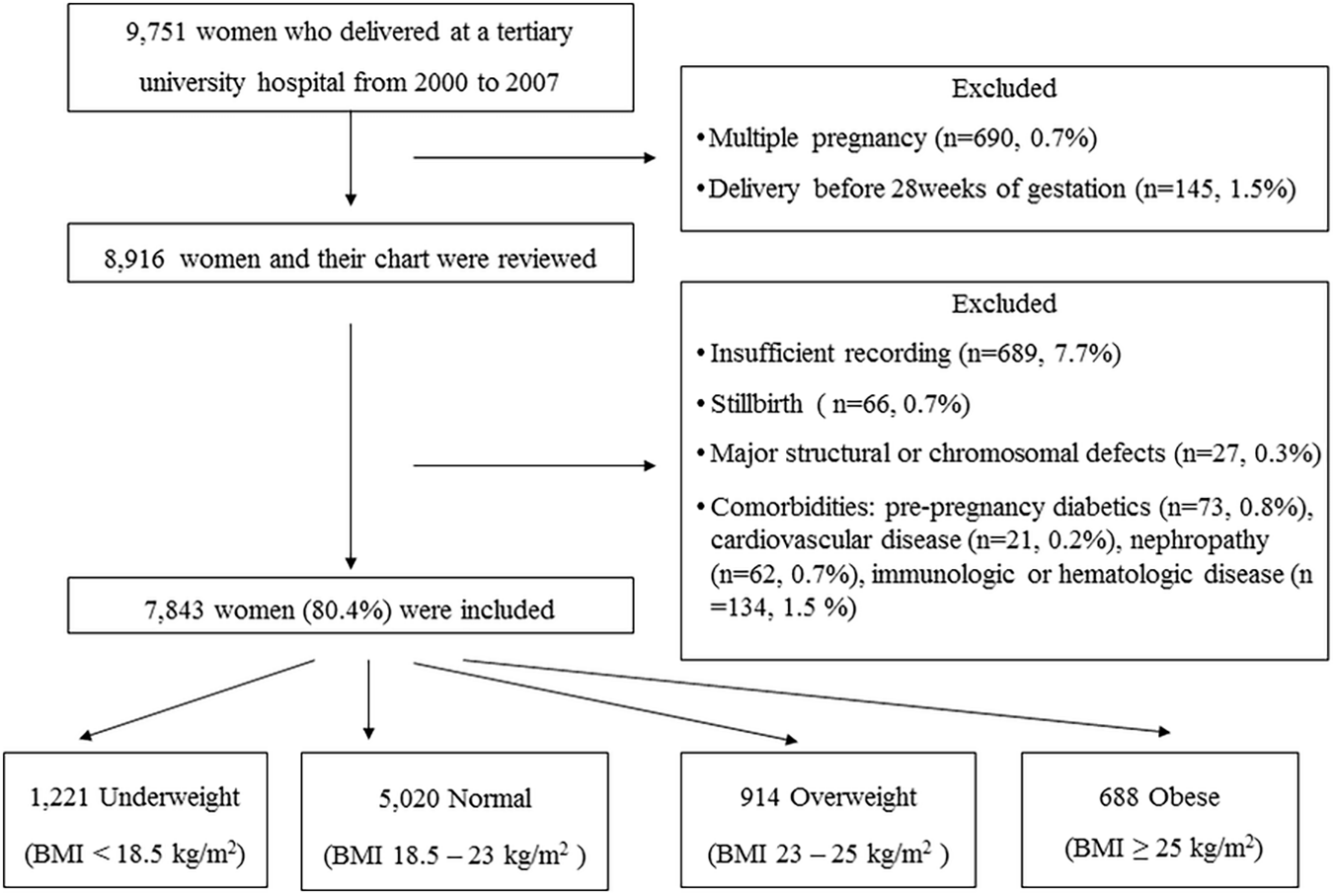
Flow diagram of study inclusion.

Individuals were excluded from the study if their medical records lack documented self-reported pre-pregnancy height and weight and weight at date of delivery, if they suffered a stillbirth, or if their neonates had major structural or chromosomal defects. Women who had pre-pregnancy diabetes, cardiovascular disease, nephropathy, or immune disease or took nonsteroidal anti-inflammatory drugs or anti-platelet drugs were excluded. Of 9,751 women who delivered during the study period, 835 women (8.6%) were excluded and 7,843 pregnant women were included in the study (Fig 1). This study was approved by the Institutional Review Board of the Catholic University of Korea.

Data were collected from the electronic medical records. Baseline data included maternal age at delivery, parity, newborn birth weight, gestational age at delivery, delivery mode, and gestational complications including gestational diabetes and preeclampsia. In addition, anthropometric data, including pre-pregnancy weight, pre-pregnancy height, and weight at delivery date were collected. Height and weight were used to calculate pre-pregnancy BMI (kg/m^2^). Pre-pregnancy anthropometric data were based on self-reported pre-pregnancy weight and height reported at the patient’s first antenatal visit to our hospital, and recorded in their chart.

All enrolled women were categorized by the Korean BMI categories recommended by the Korean Society for the Study of Obesity (KSOG) as follows: underweight, BMI of less than 18.5 kg/m^2^; normal weight, BMI of 18.5 to 22.9 kg/m^2^; overweight, BMI of 23 to 24.9 kg/m^2^; and obese, BMI of 25 kg/m^2^ or more. GWG was calculated as the difference between the maternal pre-pregnancy weight and the weight at delivery, and then classified as inadequate, optimal, or excessive based on the 2009 Institute of Medicine (IOM) recommendations [5]. The rate of weight gain per week of gestation based on the 2009 IOM recommendations were used to adjust the optimal total GWG ranges to the gestational weeks at delivery [5].

The pregnancy outcomes included SGA neonate, LGA neonate, preterm birth, preeclampsia, gestational diabetes (GDM), and cesarean delivery due to dystocia. SGA and LGA refer to neonates whose birth weight was < 10th percentile and > 90th percentile for gestational age, respectively. The Korean sex-specific reference percentiles of birth weight at each gestational age were used to classify SGA and LGA [13]. Preterm birth was defined as indicated or spontaneous birth before 37 weeks of gestation.

For statistical analysis, the baseline characteristics were compared between four groups based on pre-pregnancy BMI. In addition, baseline characteristics and pregnancy outcomes were compared between those who gained inadequate, optimal and excessive weight during pregnancy. Categorical variables were analyzed using the Chi-square test and continuous variables with one-way ANOVA. The linear-by-linear analysis test was used to evaluate linear trend among the groups. We then stratified adverse pregnancy outcomes by pre-pregnancy BMI and used a multivariate logistic regression analysis to compare women who gained excessive weight with women who gained inadequate weight. Subsequently, adjusted odds ratio (aORs) and 95% confidence intervals (CIs) for the pregnancy outcomes of interest were calculated for patients gaining inadequate or excessive weight during pregnancy after adjusting for possible confounding factors including maternal characteristics known to be related to the outcomes and pregnancy outcomes found to be significantly different from GWG groups, which include advanced maternal age, multiparity, preeclampsia and preterm birth were used for adjustment. Advanced maternal age is known to be a risk factor for preeclampsia, preterm birth, GDM, and dystocia whereas multiparity decreases the development of preeclampsia and dystocia [14–16]. Preeclampsia has been previously found to be a risk factor of SGA neonate and preterm birth [17].

The reference group in each weight gain category was weight gain within the guidelines. A *P* value of < 0.05 was considered statistically significant. SPSS (version 12.0; SPSS Inc., Chicago, IL, USA) was used for statistical analysis.

## Results

Table 1 shows the baseline characteristics and pregnancy outcomes according to pre-pregnancy BMI. Of 7,843 pregnant women, 15.6% were classified as underweight, 64.0% as normal weight, 11.7% as overweight, and 8.8% as obese based on their pre-pregnancy BMI. The prevalence of advanced maternal age was the highest in obese women. There was a significant differences between the prevalence of advanced maternal age and pre-pregnancy BMI category (*P* < 0.001) and between the distribution of multiparity and the BMI category (*P* < 0.001). In addition, the prevalence of adverse pregnancy outcomes including preterm birth, preeclampsia, gestational diabetes, and delivery of a SGA or a LGA neonate differed significantly among the pre-pregnancy BMI categories (all *P* < 0.001).

**Table 1:** Baseline characteristics and pregnancy outcomes by pre-pregnancy body mass index category.

Variable	Underweight 1221 (15.6)	Normal 5020 (64.0)	Overweight 914 (11.7)	Obese 688 (8.8)	*p* value
Maternal age (years)	30.8 ± 3.6	31.7 ± 3.7	32.3 ± 3.8	32.5 ± 3.8	< 0.001
Advanced maternal age (> 35 years)	172 (14.1)	1023 (20.4)	237 (25.9)	199 (28.9)	< 0.001
Multipara	495 (40.5)	2679 (53.34)	614 (67.2)	466 (67.7)	< 0.001
Height (cm)	161.4 ± 4.9	160.7 ± 4.9	160.2 ± 5.0	160.1 ± 5.7	<0.001
Pre-pregnancy weight (kg)	45.8 ± 8.3	53.1 ± 4.3	61.3 ± 4.0	70.3 ± 7.9	< 0.001
Pre-pregnancy BMI (kg/m^2^)	17.6 ± 0.8	20.5 ± 1.2	23.9 ± 0.6	27.4 ± 2.6	< 0.001
Preterm delivery	121 (9.9)	478 (9.5)	102 (11.2)	99 (14.4)	0.001
Preeclampsia	18 (1.5)	97 (1.9)	27 (3.0)	33 (4.8)	< 0.001
GDM	12 (1.0)	80 (1.6)	22 (2.4)	31 (4.5)	< 0.001
SGA	169 (13.8)	470 (9.4)	59 (6.5)	57 (8.3)	< 0.001
LGA	56 (4.6)	437 (8.7)	128 (14.0)	133 (19.1)	< 0.001
Cesarean delivery due to dystocia	38 / 941^a^ (4.0)	278 / 3711^a^ (7.5)	81 / 622^a^ (13.0)	58 / 425^a^ (13.6)	< 0.001

*BMI* Body mass index, *GDM* gestational diabetes, *SGA* small for gestational age, *LGA* large for gestational age. Data are expressed as number (%) or mean ± standard deviation. *p* values are from the Chi-square test or one-way ANOVA test

^a^ Number of pregnant women excluding women who underwent cesarean delivery due to other indications

For analysis of the GWG, among all of the study groups, women with adequate weight gain were the most common, followed by women with excessive weight gain and those with inadequate weight gain (42.7%, 35.6%, and 21.7%, respectively). Among underweight and normal BMI groups, almost half of women gained optimal weight, whereas the majority of women with overweight and obese BMI gained excessive weight (63.9% and 70.5% respectively). The distribution of weight gain for each BMI category is presented in Table 2. The prevalence of adverse pregnancy outcomes related with GWG showed significant differences in preterm birth, preeclampsia, SGA, LGA, and cesarean delivery due to dystocia (all *P* < 0.001 except preterm birth (*P* = 0.001)) (Table 3).

**Table 2:** Gestational weight gain according to pre-pregnancy body mass index category.

Variable	Total (N = 7843)	Underweight (N = 1221)	Normal (N = 5020)	Overweight (N = 914)	Obese (N = 688)	*p* value
Inadequate	1702 (21.7%)	434 (35.5)	1144 (22.8)	68 (7.4)	56 (8.1)	< 0.001
Adequate	3350 (42.7%)	609 (49.9)	2332 (46.5)	262 (28.7)	147 (21.4)	< 0.001
Excessive	2791 (35.6%)	178 (14.6)	1544 (30.8)	584 (63.9)	485 (70.5)	< 0.001

Data are expressed as number (%). *p* values are from the linear by linear analysis test

**Table 3:** Pregnancy outcomes by gestational weight gain among all women.

Variable	Inadequate weight gain (N = 1702)	Optimal weight gain (N = 3350)	Excessive weight gain (N = 2791)	p value
SGA	269 (15.8)	293 (8.7)	193 (6.9)	< 0.001
LGA	67 (3.9)	256 (7.6)	431 (15.4)	< 0.001
Preterm birth	198 (11.6)	294 (8.8)	308 (11.0)	0.001
Preeclampsia	12 (0.7)	45 (1.3)	118 (4.2)	< 0.001
GDM	34 (2.0)	54 (1.6)	57 (2.0)	0.403
Cesarean due to dystocia	82 / 1323^a^(6.2)	168 / 2501^a^(6.7)	205 / 1875^a^(10.9)	< 0.001

*SGA* small for gestational age, *LGA* large for gestational age, *GDM* gestational diabetes. Data are expressed as number (%). *p* values are from the Chi-square test

^a^ Number of pregnant women excluding women who underwent cesarean delivery due to other indication

After adjusting for confounding factors, for underweight women (Table 4), inadequate GWG was significantly associated with SGA (aOR 2.05; *P* < 0.001) and preterm birth (aOR 1.56; *P* = 0.035), whereas inadequate GWG had a significant negative association with LGA (aOR 0.48; *P* = 0.033) and GDM (aOR 0.26; *P* = 0.047) as compared with optimal GWG. Similarly, women with a normal BMI who gained inadequate weight were more likely to develop SGA (aOR 2.21; *P* < 0.001) and preterm birth (aOR 1.33; *P* = 0.024) and less likely to deliver a LGA neonate (aOR 0.54; *P* < 0.001) compared to women who gained optimal weight. For overweight women, inadequate GWG was significantly associated with preterm birth (aOR 2.53; *P* = 0.006). For obese women at pre-pregnancy, inadequate weight gain was not significantly associated with any adverse pregnancy outcomes.

**Table 4:** Multivariate analysis of pregnancy outcomes by pre-pregnancy body mass index category among women who gained inadequate weight during pregnancy.

Variables	Underweight aOR (95% CI)^a^	Normal aOR (95% CI)^a^	Overweight aOR (95% CI)^a^	Obese aOR (95% CI)^a^
SGA	2.05 (1.44–2.93)^d^	2.21 (1.77–2.76)^d^	0.95 (0.30–3.01)	0.69 (0.17–2.79)
LGA	0.48 (0.24–0.94)^b^	0.54 (0.39–0.75) ^d^	0.37 (0.11–1.27)	0.31 (0.07–1.41)
Preterm birth	1.56 (1.04–2.35)^b^	1.33 (1.04–1.71)^b^	2.53 (1.30–4.94)^c^	1.75 (0.80–3.85)
Preeclampsia	0.95 (0.27–3.43)	0.52 (0.23–1.20)	N/A^e^	0.30 (0.04–2.43)
GDM	0.255 (0.07–0.98)^c^	0.77 (0.44–1.37)	0.53 (0.13–2.25)	1.55 (0.41–5.78)
Cesarean due to dystocia	1.05 (0.49–2.24)	1.04(0.75–1.44)	1.57 (0.52–4.69)	1.23 (0.39–3.92)

*SGA* small for gestational age, *LGA* large for gestational age, *GDM* gestational diabetes, *aOR* adjusted odds ratio, *CI* confidence interval, *N/A* not available.

^a^ aORs obtained with a logistic regression model including preeclampsia, preterm birth, advanced maternal age (≥ 35 years), and multiparity

^b^ *p* < 0.05

^c^ *p* < 0.01

^d^ *p* < 0.001

^e^ there were no women that fit that criteria

Underweight women who gained excessive weight during pregnancy (Table 5) were more likely to develop preeclampsia (aOR 5.78; *P* = 0.003) compared to women who gained optimal weight. Women with a normal pre-pregnancy BMI who gained excessive weight were more likely to have LGA neonate (aOR 2.10; *P* < 0.001), preterm birth (aOR 1.33; *P* = 0.011), preeclampsia (aOR 2.76; *P* < 0.001), and cesarean section due to dystocia (aOR 1.37; *P* = 0.030) and less likely to deliver SGA neonate (aOR 0.60; *P* < 0.001). Overweight women at pre-pregnancy who gained excessive weight were more likely to have LGA neonate (aOR 1.56; *P* = 0.048) and preeclampsia (aOR 4.56; *P* = 0.018). Obese women at pre-pregnancy were more likely to deliver LGA neonate (aOR 2.27; *P* = 0.004) and less likely to develop GDM (aOR 0.37; *P* = 0.013) if they gained excessive weight.

**Table 5:** Multivariate analysis of pregnancy outcomes by pre-pregnancy body mass index category among women who gained excessive weight during pregnancy.

Variables	Underweight aOR (95% CI)^a^	Normal aOR (95% CI)^a^	Overweight aOR (95% CI)^a^	Obese aOR (95% CI)^a^
SGA	0.56 (0.30–1.07)	0.60 (0.46–0.78)^d^	0.82 (0.43–1.56)	0.91 (0.45–1.80)
LGA	1.23 (0.22–1.54)	2.10 (1.70–2.61)^d^	1.56 (1.05–2.55)^b^	2.27 (1.31–3.94)^b^
Preterm birth	0.98 (0.53–1.79)	1.33 (1.07–1.67)^b^	0.65 (0.40–1.06)	0.65 (0.38–1.12)
Preeclampsia	5.78(1.85–18.11)^c^	2.76 (1.75–4.36)^d^	4.56(1.29–16.07)^b^	1.17 (0.48–2.87)
GDM	0.84 (0.07–9.83)	1.19 (0.71–1.99)	0.92 (0.33–2.53)	0.37 (0.17–0.81)^b^
Cesarean due to dystocia	1.46 (0.61–3.50)	1.37 (1.03–1.81)^b^	1.65 (0.94–2.89)	0.75 (0.37–1.52)

*SGA* small for gestational age, *LGA* large for gestational age, *GDM* gestational diabetes, *aOR* adjusted odds ratio, *CI* confidence interval

^a^ aORs obtained with a logistic regression model including preeclampsia, preterm birth, advanced maternal age (≥ 35 years), and multiparity

^b^ *p* < 0.05

^c^ *p* < 0.01

^d^ *p* < 0.0011

When we used the WHO BMI criteria, for women with overweight BMI by Korean criteria, inadequate or overweight GWG by WHO BMI criteria were not significantly associated with any adverse pregnancy outcomes compared with optimal GWG (S1 Table). For women with obese BMI by Korean criteria, inadequate and excessive GWG by WHO BMI criteria were associated with delivery of LGA neonate (aOR 0.27; 95% CI 0.10–0.71 and aOR 1.55; 95% CI 1.01–2.38, respectively) (S2 Table).

## Discussion

This study showed that 64% for Korean women start their pregnancies with normal BMI. Only 15.6% of women in this study were underweight and 8.8% were obese before pregnancy. Considering that overweight and obese BMI classifications for Korean are far lower than those for Caucasian women and even lower than those for Chinese women (overweight: 24–28 kg/m^2^; obese: ≥ 28 kg/m^2^), the number of obese pregnant women in Korea was relatively low. This finding that many Korean women are in the normal BMI range before pregnancy is highly encouraging as starting pregnancy with normal BMI is associated with favorable pregnancy outcomes. Meanwhile, most adverse pregnancy outcomes such as preterm birth, preeclampsia, GDM, and delivery of a LGA neonate were found in overweight and obese women. This relationship between maternal obesity and adverse pregnancy outcomes is consistent with previous studies in other countries [18, 19].

With regard to GWG, 42.7% of the study cohort gained optimal weight based on the 2009 IOM recommendations. Almost half of women with underweight or normal BMI before pregnancy gained optimal weight within the range of IOM recommendations; however, less than 30% of overweight and obese women were compliant with the recommended IOM weight gain. The majority of these women (63.9% of the overweight group and 70.5% of the obese group) gained excessive weight.

With regard to the relationship between GWG and pregnancy outcome, it was found that excessive weight gain was generally associated with adverse pregnancy outcomes including preeclampsia, delivery of LGA neonate, and cesarean delivery due to dystocia. Although these associations were not statistically significant for some BMI categories, the trend was similar across all BMI categories. The risk of preeclampsia was increased in all women except those who were obese at pre-pregnancy. However, the sample size of obese women was too small to evaluate the effect of excessive weight gain on preeclampsia. Also, the delivery of LGA infant was significantly increased in all women except those who were underweight.

In contrast, inadequate weight gain was likely to increase the risk of delivery of a SGA neonate and preterm birth and decrease the risk of delivery of a LGA neonate. Inadequate GWG stimulates the production of epinephrine and cortisol, which stimulates maternal corticosterone-releasing-hormone and prostaglandin production. These hormones are known to be associated with preterm labor [20]. Furthermore, maternal undernutrition may be associated with impaired immunity and promotes uterine infection [21]. However, in this study, these associations were mostly found in women with underweight or normal BMI at pre-pregnancy. For overweight or obese women, the sample size was too small to ensure the effect of inadequate weight gain on pregnancy outcomes.

GWG was not associated with GDM for the overall cohort. However, when we analyze the association by pre-pregnancy BMI, underweight women who gained inadequate weight and obese women who gained excessive weight were less likely to have GDM compared with women who gained optimal weight. Previous studies have shown conflicting results regarding the GDM association with GWG. Some have suggested that the inadequate GWG is associated with GDM [10], but others have demonstrated that the GWG is not associated with GDM [22]. These conflicting results might be related to the fact that women who are diagnosed with GDM during the second trimester of pregnancy may try to maintain appropriate weight during the remainder of pregnancy by exercising and having a healthy diet. Obese women who have GDM are much more likely to be required to follow the treatment. Further study with a large number of obese women will be necessary to evaluate the relationship between GDM and GWG.

Korean pregnant women with normal BMI may be likely to decrease the development of most adverse pregnancy outcomes by adhering to GWG based on IOM recommendations. In this study, for women with normal BMI, inadequate GWG was related to preterm birth and SGA neonates. In addition, excessive GWG was related to LGA neonates, cesarean delivery due to dystocia, preeclampsia and preterm birth. Therefore, in women with normal BMI before pregnancy, it might be appropriate to follow the 2009 IOM recommendations to decrease pregnancy complications.

This study also determined that weight gain within the 2009 IOM recommendations helps increase the probability of favorable pregnancy outcomes in Korean women with underweight BMI. In fact, Asian and Caucasian women have the same BMI criteria for underweight. Thus, what this study intended to investigate was whether the optimal GWG (12.5–18 kg) recommended by the IOM in 2009 can be applied to Korean pregnant women. The results showed that weight gain of < 12.5 kg was likely to increase the delivery of a SGA neonate and preterm birth. Unlike pregnant women with normal BMI, the occurrence of LGA neonate and cesarean delivery due to dystocia was not associated with excessive GWG; only the risk of preeclampsia was increased when weight gain was excessive. This result is similar to the results from other studies conducted among Caucasian pregnant women [3]. However, in this study, the sample size of underweight women who gained excessive weight was too small to ensure the definite effect of excessive weight gain on pregnancy outcomes in underweight women.

Overweight Korean women would be classified as having normal BMI under the BMI classification for Caucasians. Thus, whether the IOM recommendations for normal BMI or those for overweight BMI should be followed is controversial. This study classified GWG based on established IOM guidelines for overweight women. Inadequate GWG was associated with a 2.5-fold greater risk for preterm birth and excessive GWG can increase the risk of delivering LGA neonate by 1.5 and that of preeclampsia by 4.6. In contrast, when overweight women followed the IOM recommendation for women with normal BMI, keeping the optimal GWG (7–11.5 kg) was unlikely to reduce any adverse pregnancy outcomes. Application of the 2009 IOM recommendations for overweight Caucasian women to overweight Korean women seems reasonable, considering the relationship between GWG and occurrence of pregnancy complications.

The criteria for obesity among Korean women (BMI ≥25 kg/m^2^) is lower than that for Caucasian women (BMI ≥30 kg/m^2^). Obese women in this study had an average BMI of 27.4 ± 2.6, which is relatively low, and only 1.1% had BMI ≥30 kg/m^2^. When we evaluated whether it is appropriate to apply the 2009 IOM standard to obese Korean women, the result showed that the delivery of a LGA neonate was likely to be lower for women who gained weight within the IOM recommendations compared with women who gained excessive weight; however, other adverse outcomes were not affected by inadequate or excessive GWG. Additionally, the IOM recommendation for women with overweight BMI seemed to affect the development of adverse pregnancy outcomes for Korean obese women.

The optimal weight gain during pregnancy for obese pregnant women has been controversial. The relationship between the occurrence of pregnancy complications and inadequate weight gain among obese women seems to be unclear. In addition, excessive GWG is related to postpartum weight retention [23]. For these reasons, several studies have argued that for obese pregnant women inadequate weight gain or even weight loss is more appropriate for their lifelong health as long as there are no complications for pregnancy [24, 25]. This study showed that optimal GWG based on IOM recommendations were not likely to be associated with favorable pregnancy outcomes. However, the sample size of obese women in this study was too small to set guidelines on optimal GWG. Further studies on a large number of obese women are necessary.

The limitations of this study include the fact that pre-pregnancy weight and height were self-reported. Practically, pre-pregnancy weight is almost based on maternal recall, because few women let their weight be measured and documented prior to conception [26]. There were reports suggesting inaccuracies in the self-reported weight and height [27, 28]. Women, especially those who are overweight or obese, tend to report a lighter than actual weight; thus, recorded GWG might have been inaccurate [28]. However, several reports supported the usefulness of self-reported height and weight [29]. A systemic review reported that reporting error did not largely bias associations between pregnancy-related weight and birth outcomes [26]. In addition, because the data were collected from hospital records from 2000 to 2007, GWG may have changed over the subsequent decade. Further studies using current data are needed. Particularly, a large number of overweight and obese women are necessary to set guidelines on optimal GWG for these women.

## Conclusion

This study showed that it is tolerable for Korean women to follow recommended GWG from the 2009 IOM guidelines to decrease pregnancy complications. This will be useful for antenatal care on GWG not only for Korean pregnant women, but also other Asian women who have lower BMI criteria than do Caucasian women. A prospective study using measured data is needed to recommend optimal weight gain for Korean women.

## Supporting information

S1 Fig Gestational weight gain according to pre-pregnancy body mass index. (TIF)

S2 Fig Pregnancy outcomes according to pre-pregnancy body mass index. (TIF)

S3 Fig Pregnancy outcomes according to gestational weight gain (GWG) for underweight pregnant women. (TIF)

S4 Fig Pregnancy outcomes according to gestational weight gain (GWG) for pregnant women with normal body mass index. (TIF)

S5 Fig Pregnancy outcomes according to gestational weight gain (GWG) for overweight pregnant women. (TIF)

S6 Fig Pregnancy outcomes according to gestational weight gain (GWG) for obese pregnant women. (TIF)

S1 Table Pregnancy outcomes by weight gain according to the IOM recommendations for women with normal or overweight pre-pregnancy body mass index among Korean overweight women. (DOCX)

S2 Table Pregnancy outcomes by weight gain according to the IOM recommendation for women with overweight or obese pre-pregnancy body mass index among Korean obese women. (DOCX)
