# Supplementary figures and images for: Is it appropriate for Korean women to adopt the 2009 Institute of Medicine recommendations for gestational weight gain?
Source: PLoS One. 2017 Jul 13;12(7):e0181164. doi: 10.1371/journal.pone.0181164 (PMC5509309; doi:10.1371/journal.pone.0181164)

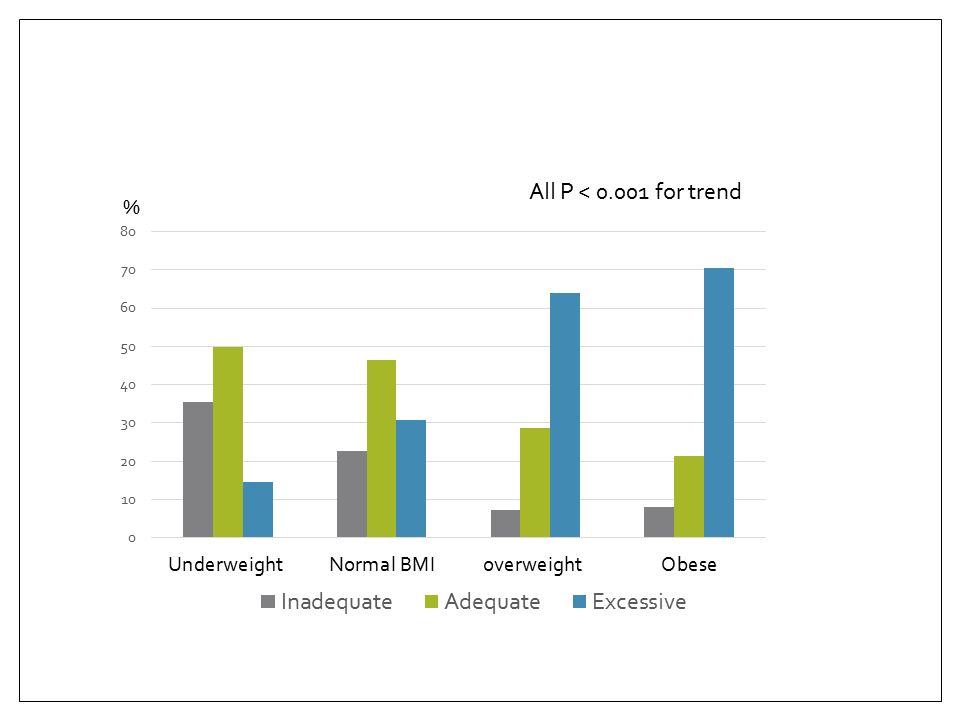

Supplement: S1 Fig — (TIF) [file pone.0181164.s001.TIF]

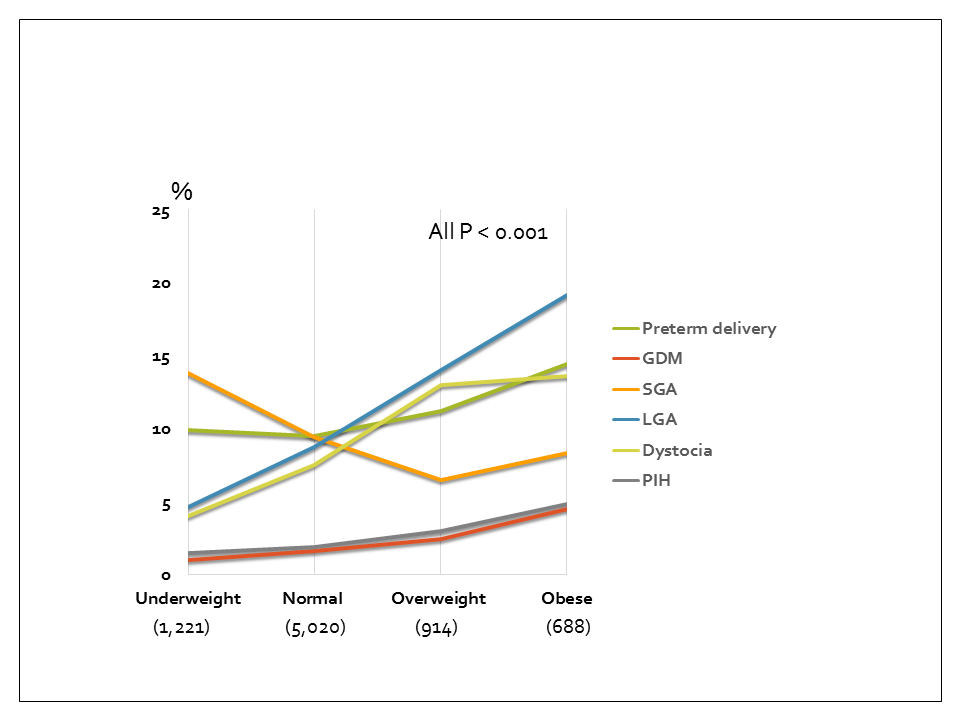

Supplement: S2 Fig — (TIF) [file pone.0181164.s002.TIF]

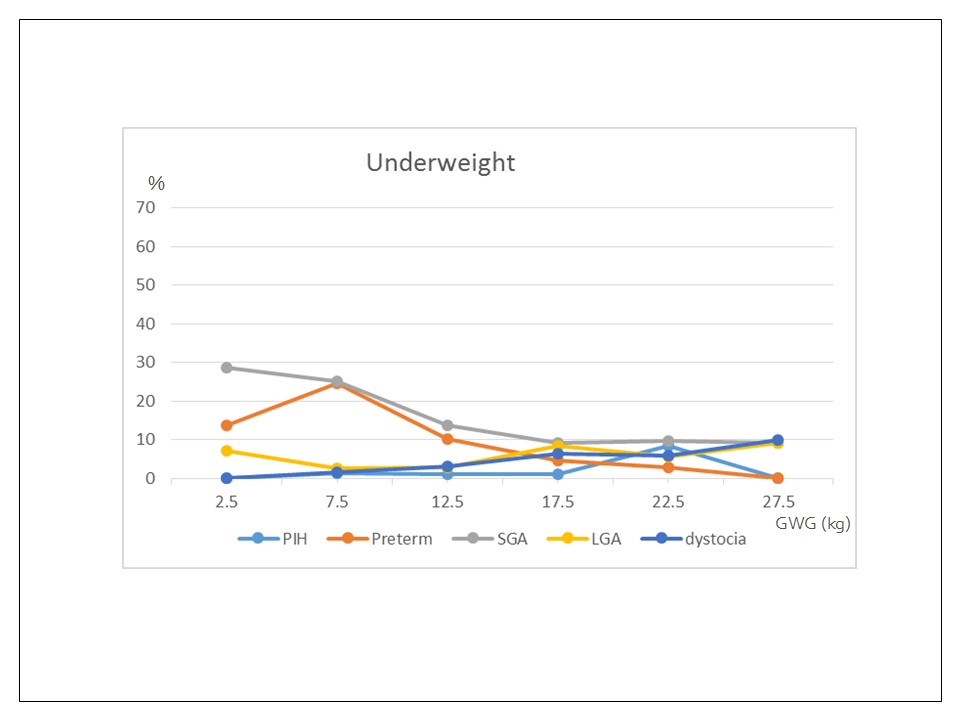

Supplement: S3 Fig — (TIF) [file pone.0181164.s003.TIF]

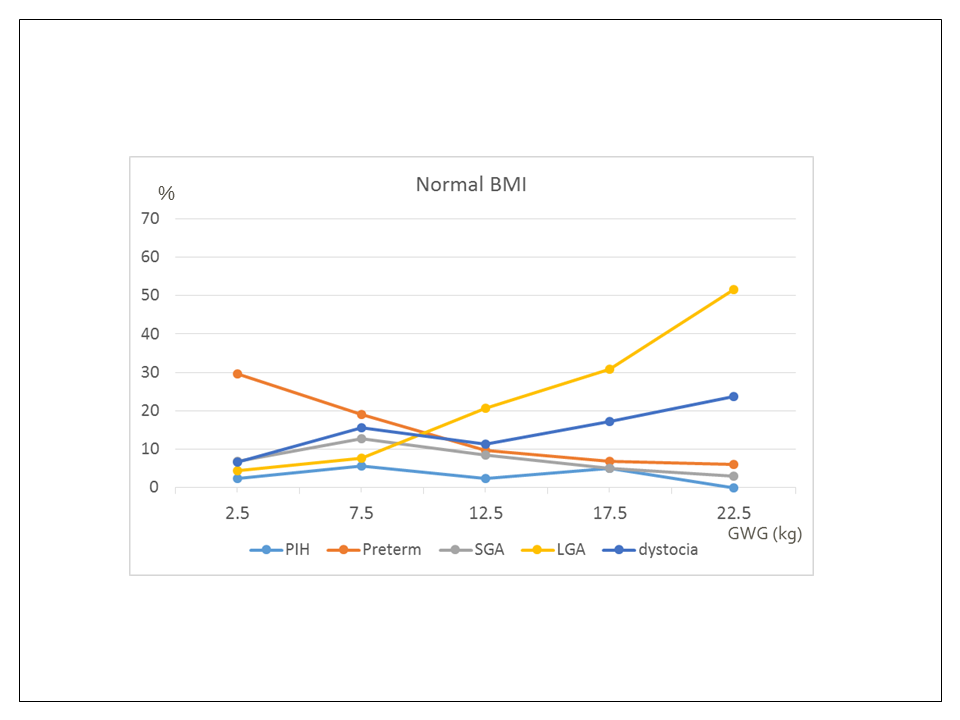

Supplement: S4 Fig — (TIF) [file pone.0181164.s004.TIF]

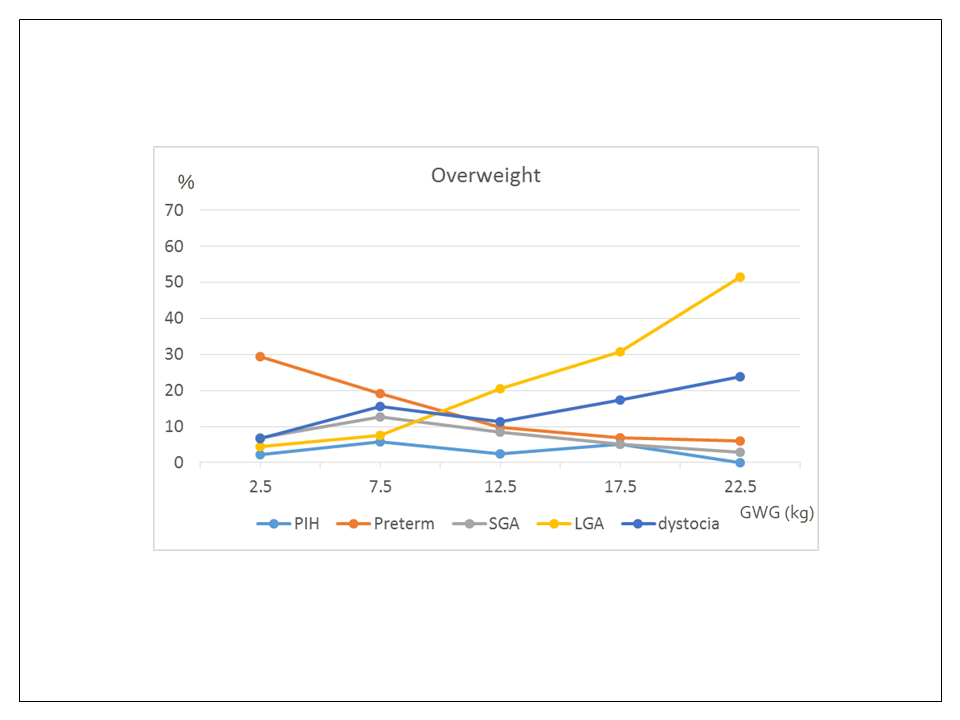

Supplement: S5 Fig — (TIF) [file pone.0181164.s005.TIF]

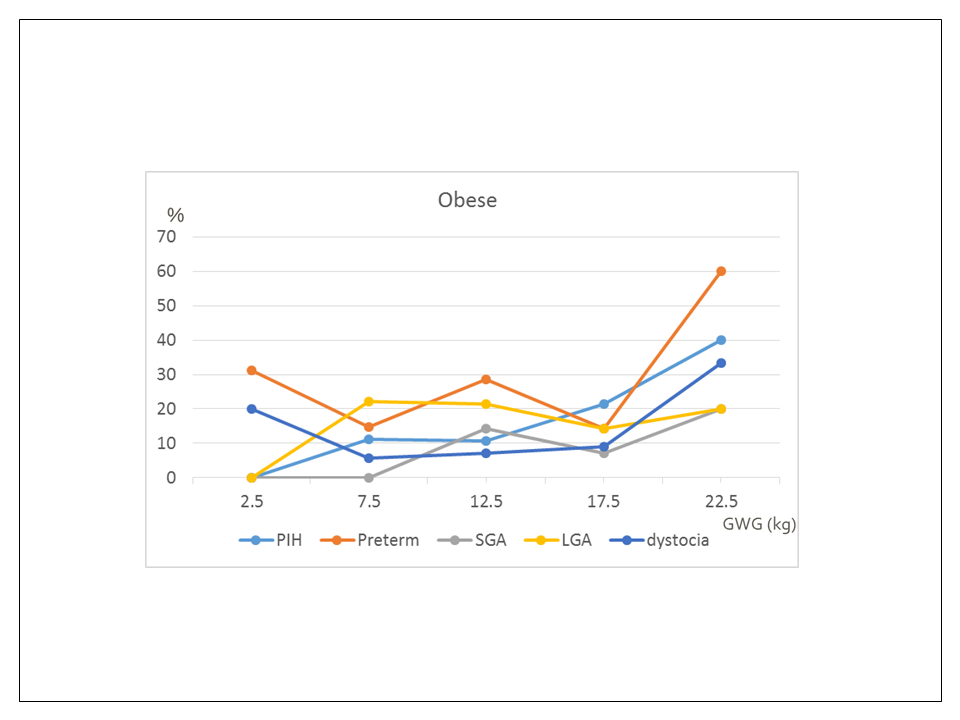

Supplement: S6 Fig — (TIF) [file pone.0181164.s006.TIF]
