# Supplementary material for: Is it appropriate for Korean women to adopt the 2009 Institute of Medicine recommendations for gestational weight gain?
Source: PLoS One. 2017 Jul 13;12(7):e0181164. doi: 10.1371/journal.pone.0181164 (PMC5509309; doi:10.1371/journal.pone.0181164)
Supplement: S1 Table — (DOCX) [file pone.0181164.s007.docx]

| Variables | IOM recommendation  for women with normal BMI | | IOM recommendation  for women with overweight BMI | |
| --- | --- | --- | --- | --- |
|  | Inadequate GWG  (< 11.5 kg) (N = 289) | Excessive GWG  ( > 16 kg ) (N = 241) | Inadequate GWG  (< 7 kg) (N = 68) | Excessive GWG  (> 11.5 kg ) (N = 584) |
| SGA | 0.91 (0.46 – 1.78) | 0.75 (0.37 – 1.51) | 0.95 (0.30 – 3.01) | 0.82 (0.43 – 1.56) |
| LGA | 0.27 (0.10 – 0.71) | 1.33 (0.85 – 2.09) | 0.37 (0.11 – 1.27) | 1.56 (1.05 – 2.55)^b^ |
| Preterm birth | 1.35 (0.82 – 2.21) | 1.18 (0.68 – 2.05) | 2.53 (1.30– 4.94)^c^ | 0.65 (0.40 – 1.06) |
| Preeclampsia | 0.35 (0.09 – 1.31) | 2.18 (0.90 – 5.27) | 0.00 (0.00 - ) | 4.56(1.29 – 16.07)^b^ |
| GDM | 0.83 (0.31 – 2.19) | 0.31 (0.08 – 1.16) | 0.53 (0.13 – 2.25) | 0.92 (0.33 – 2.53) |
| C/sec due to dystocia | 2.12 (0.79 – 5.67) | 1.66 (0.94 – 2.89) | 1.57 (0.52 – 4.69) | 1.65 (0.94 – 2.89) |

**S1 table** Pregnancy outcomes by weight gain according to the IOM recommendations for women with normal or overweight pre-pregnancy body mass index among Korean overweight women

*GWG* gestational weight gain*, SGA* small for gestational age, *LGA* large for gestational age, *BMI* body mass index

Data are expressed as adjusted odds ratio (95% confidence interval)

Adjusted odds ratios were obtained with a logistic regression model including preeclampsia, preterm birth, advanced maternal age (≥ 35 years), and multiparity

^b^ *p* < 0.05

^c^ *p* < 0.01
