# Supplementary material for: Is it appropriate for Korean women to adopt the 2009 Institute of Medicine recommendations for gestational weight gain?
Source: PLoS One. 2017 Jul 13;12(7):e0181164. doi: 10.1371/journal.pone.0181164 (PMC5509309; doi:10.1371/journal.pone.0181164)
Supplement: S2 Table — (DOCX) [file pone.0181164.s008.docx]

| Variables | IOM recommendation  for women with overweight BMI | | IOM recommendation  for women with obese BMI | |
| --- | --- | --- | --- | --- |
|  | Inadequate weight gain  (N = 100) | Excessive weight gain  (N = 372) | Inadequate weight gain  (N = 56) | Excessive weight gain  (N = 485) |
| SGA | 0.65 (0.25 – 1.67) | 0.53 (0.28 – 0.99) | 0.69 (0.17 – 2.79) | 0.91 (0.45 – 1.80) |
| LGA | 0.27 (0.10 – 0.71)**^d^** | 1.55 (1.01 – 2.38)**^c^** | 0.31 (0.07 – 1.41) | 2.27 (1.31 – 3.94)**^b^** |
| Preterm birth | 1.74 (0.35 – 1.56) | 1.02 (0.60 – 1.74) | 1.75 (0.80 – 3.85) | 0.65 (0.38 – 1.12) |
| Preeclampsia | 0.43 (0.11 – 1.72) | 1.05 (0.46 – 2.39) | 0.30 (0.04 – 2.43) | 1.17 (0.48 – 2.87) |
| GDM | 1.24 (0.46 – 3.31) | 0.56 (0.24 – 1.28) | 1.55 (0.41 – 5.78) | 0.37 (0.17 – 0.81)^b^ |
| C/sec due to dystocia | 1.23 (0.39 – 3.92) | 0.83 (0.41 – 1.66) | 1.23 (0.39 – 3.92) | 0.75 (0.37 – 1.52) |

**S2 table** Pregnancy outcomes by weight gain according to the IOM recommendation for women with overweight or obese pre-pregnancy body mass index among Korean obese women

*GWG* gestational weight gain*, SGA* small for gestational age, *LGA* large for gestational age, *BMI* body mass index

Data are expressed as adjusted odds ratio (95% confidence interval)

Adjusted odds ratios were obtained with a logistic regression model including preeclampsia, preterm birth, advanced maternal age (≥ 35 years), and multiparity

^b^ *p* < 0.05

^c^ *p* < 0.01
